# Genetic Evidence Highlights Potential Impacts of By-Catch to Cetaceans

**DOI:** 10.1371/journal.pone.0015550

**Published:** 2010-12-15

**Authors:** Martin Mendez, Howard C. Rosenbaum, Randall S. Wells, Andrew Stamper, Pablo Bordino

**Affiliations:** 1 Department of Ecology, Evolution, and Environmental Biology, Columbia University, New York, New York, United States of America; 2 Sackler Institute for Comparative Genomics, American Museum of Natural History, New York, New York, United States of America; 3 Fundación AquaMarina, Pinamar, Buenos Aires, Argentina; 4 Ocean Giants Program, Wildlife Conservation Society, Bronx, New York, United States of America; 5 Wildlife Trust Alliance, New York, New York, United States of America; 6 Chicago Zoological Society, c/o Mote Marine Laboratory, Sarasota, Florida, United States of America; 7 Walt Disney World Resorts, Lake Buena Vista, Florida, United States of America; American Museum of Natural History, United States of America

## Abstract

Incidental entanglement in fishing gear is arguably the most serious threat to many populations of small cetaceans, judging by the alarming number of captured animals. However, other aspects of this threat, such as the potential capture of mother-offspring pairs or reproductive pairs, could be equally or even more significant but have rarely been evaluated. Using a combination of demographic and genetic data we provide evidence that i) Franciscana dolphin pairs that are potentially reproductive and mother-offspring pairs form temporal bonds, and ii) are entangled simultaneously. Our results highlight potential demographic and genetic impacts of by-catch to cetacean populations: the joint entanglement of mother-offspring or reproductive pairs, compared to random individuals, might exacerbate the demographic consequences of by-catch, and the loss of groups of relatives means that significant components of genetic diversity could be lost together. Given the social nature of many odontocetes (toothed cetaceans), we suggest that these potential impacts could be rather general to the group and therefore by-catch could be more detrimental than previously considered.

## Introduction

By-catch is among the most serious threats to non-target marine fauna globally [1], estimated to impact 239 species of marine vertebrates [2] including as many as 80 out of the 85 cetacean species [3], with annual catch numbers around 300,000 cetaceans [4]. Cetacean by-catch is particularly serious given the high sociality [5], slow life histories and limited potential for population growth in these species [6]. Despite the known direct impacts of by-catch on cetacean abundance, there is little knowledge about its potential impacts to specific social groups, such as mother-offspring pairs and reproductive pairs. From a demographic perspective, mother-offspring and reproductive pairs have a crucial effect on the population persistence, given that these individuals have relatively high reproductive values [7], [8], [9]. From a genetic perspective, family groups represent an important component of the intra-population genetic diversity, which is associated with the potential of a population to withstand environmental variation [10], [11], [12].

We seek to evaluate potential impacts of by-catch to specific family groupings in cetaceans, focusing on the rare Franciscana dolphin (*Pontoporia blainvillei*), endemic to the Western South Atlantic Ocean and possibly the most impacted cetacean in the region [13]. Incidental catches are estimated at a minimum of 3000 Franciscanas annually along the entire species' distribution range, from southern Brazil through northern Argentina [14], [15]. These data coupled with abundance estimations suggest that, annually, by-catch alone is removing a minimum of 3% of the population in some areas in Brazil [16] and between 2% and 5% of the population in Argentina [14], [17]. However, there are no data on the impact of by-catch to specific demographic associations within these populations in Argentina. The only published account of by-catch to Franciscana social groups was for a group of 4 animals simultaneously entangled in Brazilian waters, known to represent a distinct Franciscana population to those found in Argentina [18], [19], [20].

Here we use a combination of field, demographic and genetic data to evaluate the potential impact of by-catch to important demographic associations of cetaceans. Specifically, we focus on dolphin pairs that showed evidence of spatial association, and pairs that have been simultaneously by-caught, and investigate whether these animals are part of the same family group (i.e. mother-offspring, siblings, etc.), reproductive group, or are unrelated individuals. This knowledge will inform us about potential demographic and genetic impacts to Franciscana dolphins, and possibly other social cetaceans.

## Methods

**Figure 1:**
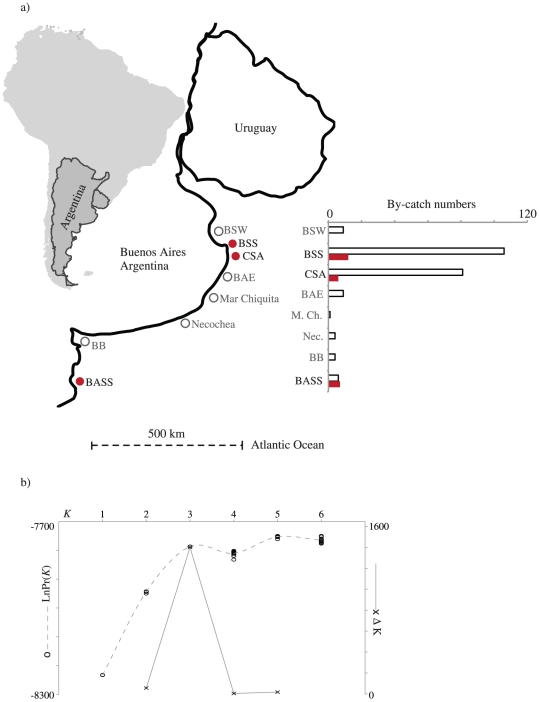
Study area and sampling effort. a) Study area map showing the frequency distribution of individual (empty bars) and simultaneous (solid red bars) incidental entanglements/capture release events in the Buenos Aires province, with the locations of the simultaneous events highlighted in red color. b) Population structure between BSS, CSA and BASS (Table S3). The graph displays the Log-likelihood of the data and the *ΔK*, plotted against the partition number.

Tissue samples from 245 individuals were obtained mostly from incidentally entangled Franciscana dolphins in fishing gear in Argentina during 2000 through 2009. At least 14 dolphins were by-caught simultaneously, in pairs in the same net, in Bahia Samborombon South (BSS) and Cabo San Antonio (CSA) (Figure 1a). In addition, four pairs and a group of three individuals were captured for tagging and released during 2006 through 2008 in locations BSS and Bahia San Blas (BASS) – the individuals of each of these five groups were swimming together at the time of capture [21] (Figure 1a). Dolphin tagging and tissue sampling work for this study was undertaken after approval by the “Dirección de Areas Protegidas y Conservación de la Biodiversidad (Buenos Aires Government)”, and under scientific research permits N° 50/04, 01/06, 01/07, and 01/08. We recorded sex, body length and condition for all simultaneously entangled or captured-released individuals (Table 1).

**Table 1:** Demographic and genetic information of the captured and released groups and simultaneously entangled animals.

Population	Status	Goup #	Individual code	LT (cm)	Age	Gender	mtDNA hap
BSS	by-catch	1	Pb_SC_05_047	129	adult	F	1
BSS	by-catch	1	Pb_SC_05_048	89	calf	F	1
BSS	by-catch	2	Pb_SC_05_065	134	adult	F	4
BSS	by-catch	2	Pb_SC_05_066	98.5	calf	M	4
BSS	by-catch	3	Pb_SC_05_073	84	calf	F	1
BSS	by-catch	3	Pb_SC_05_074	127	adult	F	1
BSS	by-catch	4	Pb_SC_05_084	125	adult	M	4
BSS	by-catch	4	Pb_SC_05_085	74.5	calf	M	1
BSS	tagged	5	Pb_SCcap_06_1	147	adult	F	6
BSS	tagged	5	Pb_SCcap_06_2	115	adult	M	7
BSS	tagged	6	Pb_SCcap_06_3	147	adult	F	1
BSS	tagged	6	Pb_SCcap_06_4	130	adult	M	8
CSA	by-catch	7	Pb_MA_05_010	150	adult	F	3
CSA	by-catch	7	Pb_MA_05_011	120	adult	M	4
CSA	by-catch	8	Pb_MA_04_075	>100	adult	F	1
CSA	by-catch	8	Pb_MA_04_076	>100	adult	M	2
CSA	by-catch	9	Pb_SB_04_054	128	adult	F	4
CSA	by-catch	9	Pb_SB_04_055	117.2	adult	M	4
BASS	tagged	10	Pb_SBScap_07_3	140	adult	M	5
BASS	tagged	10	Pb_SBScap_07_4	142	adult	F	4
BASS	tagged	11	Pb_SBScap_08_1	128	adult	M	6
BASS	tagged	11	Pb_SBScap_08_2	147	adult	F	4
BASS	tagged	11	Pb_SBScap_08_3	105	calf	F	4
BASS	tagged	12	Pb_SBScap_08_4	132	adult	M	5
BASS	tagged	12	Pb_SBScap_08_5	147	adult	F	4

To investigate the genealogical relationships between all simultaneously entangled and captured dolphins, we extracted genomic DNA, confirmed visual sexing with molecular techniques, sequenced a 560 bp mitochondrial DNA (mtDNA) fragment in the control region, and genotyped all samples using 12 microsatellite markers optimized for this species (Table S1). All genetic laboratory procedures are described in detail and published elsewhere [19], [20].

For the microsatellite data, GENEPOP v4.0 [22] was used to evaluate linkage disequilibrium (LD) between all pairs of loci for each population (1000 dememorization iterations, 1000 batches, 10000 iterations per batch) and Hardy-Weinberg equilibrium (HWE). Significance levels (*p* = 0.05) for departure from HWE and for LD were corrected for multiple comparisons with the sequential Bonferroni correction [23]. Population structure assessments are needed prior to relatedness estimations, as genetic partitioning can influence such estimations [24]. We used mtDNA and microsatellite data to evaluate population structure between the three sites where the multiple entanglements and capture-release operations took place (BSS, CSA and BASS). Spatial structure of the mitochondrial dataset was evaluated through an estimation of pairwise *F_ST_* (haplotype frequencies only) and *Φ_ST_* statistics (using the Kimura 2-parameter correction), computed using Arlequin v3.1[25], [26]. The significance of the observed *Φ*- or *F*-statistics was tested using the null distribution generated from 10,000 non-parametric random permutations of the data. This estimation was done between BSS-CSA and between CSA-BASS, given the coastal habits of these dolphins and that the three sampling sites are separated along the same coastline. In addition, we assessed the degree of partitioning in our total sample without *a priori* definition of putative populations using a Bayesian clustering algorithm on the microsatellite data with STRUCTURE v2.3.1 [27]. We used the admixture model, which assumes that individuals have mixed ancestry, and did not include sampling origin information in our priors, making our model more stringent. We performed 10 independent long runs (10^6^ burn-in steps, 10^7^ total steps) for *each* value of *K* (1≤*K*≤6), for a total of 60 runs (Table S2), and assessed convergence through the observation of the ALPHA value for each run. The output of the Bayesian runs was interpreted via a heuristic approach and following the Δ*K* approach [28]. Further details of the analysis of population structure are provided in the Supporting Information section.

Pedigree relationships were evaluated with KINGROUP v2.0.8. [29]. Relatedness estimations for each pair of simultaneously entangled or captured-released dolphins were performed within their respective population of origin, identified with the previous analyses of population structure. First, we evaluated the performance of the most commonly used relatedness estimators for our dataset, rQG [30], rLR [31], rW [32], and rML [29] by assessing sample mean and variances of simulated relatedness measures for known relationships [33]. We then used the best performing estimators to calculate relatedness coefficients for all pairs of individuals simultaneously entangled or captured, to be able to infer genealogical relationships. Finally, we evaluated the appropriateness of a likelihood ratio approach to alternative pedigree hypotheses with a simulation exercise. Briefly, we simulated alternative scenarios of allele frequencies and pairs of individuals of the relationship we sought to test, and assessed the type II error of the likelihood ratio tests. Given the high statistical power needed for these tests, some samples present a high percentage of type II error, whereby individuals of a certain relationship would not be resolved as such due an insufficient number of alleles and/or loci in the sample. We assessed the type II error of the likelihood ratio tests using PO as the primary (alternative) hypothesis and U, HS or FS as null hypotheses. We repeated this simulation and assessment procedure ten times for each population.

## Results

Four of the seven pairs of simultaneously by-caught dolphins consisted of an adult and a juvenile dolphin. In three out of these four pairs (groups 1, 2, and 3) the adult dolphin was a female and both dolphins had the same mtDNA haplotype. The fourth pair was composed of an adult male and a juvenile male with different mtDNA haplotypes. The other three by-caught pairs were adult female-male pairs (groups 7, 8, and 9); one of them with individuals sharing their mtDNA haplotype. All of the captured and released pairs consisted of an adult female and an adult male with different mtDNA haplotypes, whereas the trio (group 11) consisted of an adult female and male with different haplotypes and a calf that shared her haplotype with the adult female (Table 1).

We found significant mtDNA structure between BSS and CSA (*F_ST_*  = 0.054, *p*<0.001; *Φ_ST_*  = 0.068, *p* = 0.031), and less significant structure between CSA and BASS (*F_ST_*  = 0.138, *p*<0.001; *Φ_ST_*  = 0.026, *p* = 0.165). The microsatellite data showed no evidence of HW disequilibrium or LD, the STRUCTURE runs showed convergence and were concordant with a hypothesis of three genetic partitions, evidenced by the plateau in the log likelihood values at *K* = 3, also coincident with the maximum Δ*K* for *K* = 3 (Figure 1 and Table S3). We therefore carried out relatedness calculations for each population independently.

Performance of the relatedness estimators was consistent between categories within populations and relatively consistent between populations: the maximum likelihood estimator r_ML_ performed best for all categories across all populations, r_LR_ performed worst for BSS and CSA, r_W_ performed worst for BASS, and r_QG_ showed intermediate performance. In seven out of the 48 tests the estimators deviated from expected values: r_LR_ deviated three times, r_W_ deviated twice, and r_QG_, and r_ML_ deviated only once (Table 2). We therefore ranked the relatedness estimators in decreasing order of overall performance as follows: r_ML_, r_QG_, r_W_, and r_LR_, and discarded r_LR_ for our subsequent analyses.

**Table 2:** Mean relatedness (m) and standard deviation (*SD*) for the 100 simulated pairs of individuals of each genealogical relationship, based on the BSS, CSA and BASS allele frequencies.

	Relationship											
	PO r_T_ = 0.5			FS r_T_ = 0.5			HS r_T_ = 0.25			U r_T_ = 0		
	m	SD	*P*	m	SD	*P*	m	SD	*P*	m	SD	*P*
**BSS**												
** r_QG_**	0.477	0.094	**0.020**	0.506	0.159	0.700	0.245	0.168	0.777	0.009	0.175	0.594
** r_LR_**	0.463	0.157	**0.022**	0.521	0.209	0.316	0.294	0.209	**0.037**	0.011	0.120	0.349
** r_W_**	0.486	0.073	0.075	0.526	0.162	0.104	0.265	0.156	0.318	0.013	0.177	0.460
** r_ML_**	0.483	0.064	**0.010**	0.531	0.150	0.037	0.274	0.147	0.100	0.024	0.130	0.063
**CSA**												
** r_QG_**	0.488	0.094	0.816	0.482	0.137	0.203	0.249	0.154	0.957	−0.001	0.149	0.975
** r_LR_**	0.490	0.141	0.503	0.483	0.151	0.268	0.253	0.187	0.853	−0.002	0.106	0.810
** r_W_**	0.497	0.073	0.758	0.489	0.129	0.399	0.237	0.163	0.448	−0.011	0.153	0.438
** r_ML_**	0.502	0.063	0.647	0.499	0.125	0.975	0.243	0.148	0.674	0.011	0.092	0.209
**BASS**												
** r_QG_**	0.501	0.100	0.857	0.480	0.159	0.221	0.243	0.151	0.658	0.013	0.173	0.446
** r_LR_**	0.460	0.136	**0.005**	0.473	0.173	0.134	0.237	0.143	0.383	0.003	0.139	0.778
** r_W_**	0.518	0.257	0.475	0.555	0.288	0.055	0.479	0.199	**0.001**	0.425	0.184	**0.001**
** r_ML_**	0.499	0.077	0.917	0.504	0.138	0.771	0.251	0.142	0.930	0.020	0.136	0.141

*P* values <0.05 show significant departures from the expected (theoretical) relatedness values (r_T_ = 0.5; r_T_ = 0.25; r_T_ = 0).

The three relatedness estimators utilized to assess our sample show consistent results: pairs 1, 2, 3 and 11 are significantly related (p_r est_<0.05) and display relatedness values r_est_ ∼0.5, whereas all other pairs are not significantly related (p_r est_>0.05) and all display relatedness values r_est_∼0, with the exception of individuals 1 and 2 in pair 11 (r_est_∼0.2) (Table 3). Likelihood ratio tests to further evaluate the hypothesis of a PO relationship for pairs 1, 2, 3 and 11, proved inappropriate for our dataset, given our simulations. Specifically, although the simulated scenarios under assumptions of equidistant, triangular and random allele frequency distributions resulted in acceptable type II error rates between 2.8% and 8.4%, those simulations utilizing our sample allele frequencies resulted in type II error rates between 25% and 29%, which translate into rejecting a PO relationship when this relationship is actually true in 25–29% of the cases, assuming a *p-value* of 0.05 (Table S4).

**Table 3:** Estimated relatedness for all groups of individuals.

Population	Status	Group #	Relationship code	rML	p (rML)	rQG	p (rQG)	rW	p (rW)
BSS	by-caught	1	SC_05_47/48	0.489	**0.005**	0.511	**0.009**	0.491	**0.004**
BSS	by-caught	2	SC_05_65/66	0.421	**0.016**	0.449	**0.024**	0.42	**0.012**
BSS	by-caught	3	SC_05_73/74	0.39	**0.023**	0.49	**0.012**	0.395	**0.017**
BSS	by-caught	4	SC_05_84/85	−0.111	0.945	−0.225	0.986	−0.439	0.998
BSS	tagged	5	SCcap_06_1/2	−0.146	0.976	0.085	0.585	−0.22	0.885
BSS	tagged	6	SCcap_06_3/4	0.118	0.353	0.198	0.322	0.15	0.205
CSA	by-caught	7	MA_05_10/11	0.026	0.415	−0.11	0.747	0.058	0.301
CSA	by-caught	8	MA_04_75/76	0.102	0.202	0.024	0.36	−0.093	0.763
CSA	by-caught	9	SB_04_54/55	−0.062	0.819	−0.309	0.993	−0.148	0.891
BASS	tagged	10	SBScap_07_3/4	−0.024	0.663	0.086	0.286	−0.099	0.731
BASS	tagged	11	SBScap_08_1/2	0.212	0.074	0.184	0.108	0.079	0.266
BASS	tagged	11	SBScap_08_2/3	0.456	**0.001**	0.356	**0.008**	0.251	**0.035**
BASS	tagged	11	SBScap_08_1/3	0.038	0.47	0.099	0.257	−0.069	0.654
BASS	tagged	12	SBScap_08_4/5	−0.077	0.943	0.068	0.332	−0.02	0.522

*P* values <0.05 show significant relationships (in bold).

## Discussion

Our combined evidence suggests that small family groups of Franciscana dolphins involving mature females swim together and are by-caught simultaneously along the Argentinean coast. Specifically, our genetic data suggest that mother-offspring associations (pair 11) and unrelated adult pairs that are potentially reproductive (pairs 5, 6, 10, 12 and adults in pair 11) were captured-released together, suggesting that they form temporary (or long term) bonds, and are therefore at risk of simultaneous entanglement. Mother-offspring pairing is well known for cetaceans, and female-male pairing among adult dolphins is typically attributed to reproduction, in agreement with our suggestions that these adult pairs could be reproductive associations [34]. We also show that mother-offspring pairs (groups 1, 2 and 3), and pairs that are potentially reproductive (pairs 7, 8, and 9) are by-caught by local fisheries, providing evidence to sustain our presumption of by-catch risk. Although we cannot be certain that all adult pairs (captured and released or by-caught) are mating, the fact that animals in these pairs have different mitochondrial haplotypes and are not significantly related according to the microsatellite data rules out the possibility of adult males swimming with their mothers. Although adult male-mother pairing is uncommon for cetaceans, it has been observed in killer whales (*Orcinus orca*) and pilot whales (*Globicephala melas*) [35], [36]. Our data also rule out any other family associations for these adult pairs, such as sibling or first cousin relationships, swimming or being entangled together. In the absence of any evidence suggesting that the adult pairs in our sample are part of the same family group, our presumption of reproductive pairs seems the most plausible cause for such pairing. Other examples of cetacean family groups traveling together [35], including Franciscana dolphins [18], support our general findings and highlight the significance of this threat to other cetaceans.

We have not observed a gender bias in the chance of by-catch for adult individuals or pairs of individuals in any of our study areas or the region as a whole (*p_X_2*>0.05; *p_Fisher_>0.05* for both BSS and CSA and the entire region). What our data suggest is that the *consequences* of multiple entanglements could be quite serious when pairs that are potentially reproductive and mother-offspring pairs are lost together, since they contribute more significantly to the population growth rate and persistence than random individuals [37].

From the ‘first principles’ of demography, it is well established that the juvenile and adult survival elasticities (the proportional change in population growth rate as a function of a proportional change in a demographic transition) are typically high for long-lived species such as marine mammals [38], [39]. Supporting this theoretical prediction, increased mortality of mothers has been attributed to the marked declines in population growth rate and life expectancy for North Atlantic right whales (*Eubalaena glacialis*) [9]. In addition, the importance of juvenile survival was empirically demonstrated for a small cetacean, the harbor porpoise (*Phocoena phocoena*), through Bayesian modeling approaches [40]. Lastly, the establishment of reproductive pairs is essential for the realization of the female's reproductive potential [41], [42]. Therefore, within the franciscana dolphin by-catch we see demographic elements (i.e. mothers, juveniles and reproductive pairs) that contribute most to reproductive output, fecundity, life expectancy and population growth rate, and that therefore provide the potential for population recovery.

From a genetic perspective, harvest will inevitably change the make up of impacted populations (i.e. their genetic diversity and effective population size) and their relationship with other populations (i.e. population subdivision parameters) [11]. When the harvest, or by-catch in our case, impacts family groups (i.e. mother-offspring pairs), the loss of genetic diversity and alteration of inter-population structure might be exacerbated by genetic drift [43]. As both genetic diversity and population structure play roles in the potential for local adaptation [44], [45], the loss of family groups is also concerning for population persistence from a genetic standpoint.

We realize that our finding of mother-offspring pairs and adult pairs forming temporal bonds and being by-caught simultaneously does not directly translate into an evaluation of the impact of loosing these social groupings on the population persistence. Rather, our data highlight relevant genetic and demographic aspects associated with by-catch, which may have been previously overlooked, and that could *potentially* add to the known depletion impact of by-catch. Whether the loss of mother-offspring and reproductive pairs actually does result in an “extra” impact to the depletion effect of by-catch could be evaluated through demographic and genetic modeling, which is a next step on our research efforts.

There appears to be some geographic segregation to the types of associations found in our study, although this is not conclusive due to the small sample size. Most mother-offspring pairs were documented in BSS and no records were obtained in CSA. Coupled with recent evidence of strong population structure between BSS and other sites in Argentina [20], a relatively high proportion of adult-calf sightings in BSS, and high fish biomass in the area [46], these data support previous suggestions that BSS could be a nursing or calving ground for the species [20]. Because by-catch events have been shown to be spatially and temporally clustered [47], a potential situation of high by-catch rates in a breeding area would be particularly serious.

Our data provides evidence that mother-offspring and reproductive pairs of Franciscanas are impacted by by-catch, and highlight potential synergies from genetic and demographic impacts on this and possibly other social cetaceans. On the one hand, by-catch removes large numbers of individuals from their populations, which is in itself a serious demographic and genetic impact [4], [11], [37]. On the other hand, mother-offspring associations and reproductive pairs are also inordinately impacted, which could exacerbate the demographic and genetic consequences of decline and possibly limiting potential for population recovery [7], [9].

Although the potential impacts of by-catch are surely manifold and difficult to quantify, we believe that our approach combining field, demographic and genetic evidence can provide a more comprehensive picture of this threat than estimating the number of by-caught animals alone. Moreover, including population structure evaluations as part of a threat assessment strategy seems particularly relevant for highly mobile species, given the potential for population connectivity across large marine areas. One of the caveats when using genetic data to make kinship inferences is that the data need to show enough variability to allow statistical testing under some frameworks. In our example, the magnitude of the type II error rates we observe in the likelihood ratio tests with our empirical data is likely a consequence of a relatively low number of loci and/or alleles, or a relatively small sample size of our empirical data. To test this, we have used KINGROUP to run simulations with higher numbers of alleles, loci and bigger population sizes, and in fact observed a marked reduction in the type II error rates (data not shown).

While we base our assumptions of demographic and genetic consequences of mother-offspring and reproductive pair by-catch on what are general principles in demography, modeling exercises using empirical data would certainly contribute to this issue, and are among the next steps in our research efforts.

## Supporting Information

Table S1 Microsatellite amplification conditions.
(DOC)

Table S2 Runs for the Bayesian analysis of population structure. Burnin steps maximize the chances of reaching a high probability region in the probability space before the actual estimation.
(DOC)

Table S3 Runs for the Bayesian analysis of population structure. Burnin steps maximize the chances of reaching a high probability region in the probability space before the actual estimation.
(DOC)

Table S4 Runs for the Bayesian analysis of population structure. Burnin steps maximize the chances of reaching a high probability region in the probability space before the actual estimation.
(DOC)

Text S1 Supporting Text.
(DOC)
