# Supplementary material for: Genetic Evidence Highlights Potential Impacts of By-Catch to Cetaceans
Source: PLoS One. 2010 Dec 15;5(12):e15550. doi: 10.1371/journal.pone.0015550 (PMC3002289; doi:10.1371/journal.pone.0015550)
Supplement: Table S1 — Microsatellite amplification conditions. (DOC) [file pone.0015550.s001.doc]

| **Marker** | **nC 1** | **D1 (°C)** | **D1 (s)** | **A1 (°C)** | **A1 (s)** | **E1 (°C)** | **E1 (s)** | **nC 2** | **D2 (°C)** | **D2 (s)** | **A2 (°C)** | **A2 (s)** | **E2 (°C)** | **E2 (s)** | **Origin** |
| --- | --- | --- | --- | --- | --- | --- | --- | --- | --- | --- | --- | --- | --- | --- | --- |
| EV5 | 10 | 92 | 30 | 60* | 1C D/c | 72 | 60 | 24 | 92 | 30 | 56 | 30 | 72 | 60 | Valsecchi *et al.* (1996) |
| EV76 | 10 | 92 | 30 | 58* | 1C D/s | 72 | 60 | 24 | 92 | 30 | 56 | 30 | 72 | 60 | Valsecchi *et al.* (1996) |
| EV104 | 10 | 92 | 30 | 54* | 1C D/s | 72 | 60 | 24 | 92 | 30 | 56 | 30 | 72 | 60 | Valsecchi *et al.* (1996) |
| D14 | 10 | 92 | 30 | 56* | 1C D/s | 72 | 60 | 24 | 92 | 30 | 56 | 30 | 72 | 60 | Shinohara *et al.* (1997) |
| D22 | 10 | 92 | 30 | 60* | 1C D/s | 72 | 60 | 24 | 92 | 30 | 56 | 30 | 72 | 60 | Shinohara *et al.* (1997) |
| FB2 | 35 | 94 | 30 | 54 | 45 | 72 | 45 | 15 | 94 | 30 | 53 | 45 | 72 | 45 | Buchanan *et al.* (1996) |
| FB5 | 35 | 94 | 30 | 58 | 45 | 72 | 45 | 15 | 94 | 30 | 53 | 45 | 72 | 45 | Buchanan *et al.* (1996) |
| FB17 | 35 | 94 | 30 | 60 | 45 | 72 | 45 | 15 | 94 | 30 | 53 | 45 | 72 | 45 | Buchanan *et al.* (1996) |
| MK5 | 10 | 92 | 30 | 50* | 1C D/s | 72 | 60 | 24 | 92 | 30 | 56 | 30 | 72 | 60 | Krutzen *et al.* (2001) |
| MK6 | 10 | 92 | 30 | 50* | 1C D/s | 72 | 60 | 24 | 92 | 30 | 56 | 30 | 72 | 60 | Krutzen *et al.* (2001) |
| MK8 | 10 | 92 | 30 | 56* | 1C D/s | 72 | 60 | 24 | 92 | 30 | 56 | 30 | 72 | 60 | Krutzen *et al.* (2001) |
| WK12 | 10 | 92 | 30 | 50* | 1C D/s | 72 | 60 | 24 | 92 | 30 | 56 | 30 | 72 | 60 | Hoelzel *et al.* (1998) |

Table S1
