# Supplementary material for: Genetic Evidence Highlights Potential Impacts of By-Catch to Cetaceans
Source: PLoS One. 2010 Dec 15;5(12):e15550. doi: 10.1371/journal.pone.0015550 (PMC3002289; doi:10.1371/journal.pone.0015550)
Supplement: Table S2 — Runs for the Bayesian analysis of population structure. Burnin steps maximize the chances of reaching a high probability region in the probability space before the actual estimation. (DOC) [file pone.0015550.s002.doc]

| **k** | **Burnin** | **# of cycles** | **LnP(D)** | **SD** | **alpha** | **Run** |
| --- | --- | --- | --- | --- | --- | --- |
| 1 | 1x10^6^ | 1x10^7^ | -8233 | 7.176 | na | 1 |
| 1 | 1x10^6^ | 1x10^7^ | -8233 | 7.169 | na | 2 |
| 1 | 1x10^6^ | 1x10^7^ | -8233.3 | 7.204 | na | 3 |
| 1 | 1x10^6^ | 1x10^7^ | -8233.1 | 7.190 | na | 4 |
| 1 | 1x10^6^ | 1x10^7^ | -8233.1 | 7.176 | na | 5 |
| 1 | 1x10^6^ | 1x10^7^ | -8233.1 | 7.183 | na | 6 |
| 1 | 1x10^6^ | 1x10^7^ | -8233.1 | 7.190 | na | 7 |
| 1 | 1x10^6^ | 1x10^7^ | -8233 | 7.169 | na | 8 |
| 1 | 1x10^6^ | 1x10^7^ | -8233.2 | 7.190 | na | 9 |
| 1 | 1x10^6^ | 1x10^7^ | -8232.7 | 7.134 | na | 10 |
| 2 | 1x10^6^ | 1x10^7^ | -7942.8 | 15.473 | 0.187 | 11 |
| 2 | 1x10^6^ | 1x10^7^ | -7943 | 15.479 | 0.186 | 12 |
| 2 | 1x10^6^ | 1x10^7^ | -7942.5 | 15.456 | 0.187 | 13 |
| 2 | 1x10^6^ | 1x10^7^ | -7949.8 | 15.881 | 0.185 | 14 |
| 2 | 1x10^6^ | 1x10^7^ | -7943.6 | 15.515 | 0.186 | 15 |
| 2 | 1x10^6^ | 1x10^7^ | -7942.8 | 15.479 | 0.185 | 16 |
| 2 | 1x10^6^ | 1x10^7^ | -7944.1 | 15.543 | 0.187 | 17 |
| 2 | 1x10^6^ | 1x10^7^ | -7944.1 | 15.550 | 0.184 | 18 |
| 2 | 1x10^6^ | 1x10^7^ | -7942.2 | 15.440 | 0.187 | 19 |
| 2 | 1x10^6^ | 1x10^7^ | -7943.1 | 15.485 | 0.186 | 20 |
| 3 | 1x10^6^ | 1x10^7^ | -7786.6 | 17.085 | 0.074 | 21 |
| 3 | 1x10^6^ | 1x10^7^ | -7786.4 | 17.079 | 0.074 | 22 |
| 3 | 1x10^6^ | 1x10^7^ | -7786.5 | 17.076 | 0.074 | 23 |
| 3 | 1x10^6^ | 1x10^7^ | -7786.3 | 17.070 | 0.074 | 24 |
| 3 | 1x10^6^ | 1x10^7^ | -7786.6 | 17.085 | 0.075 | 25 |
| 3 | 1x10^6^ | 1x10^7^ | -7786.4 | 17.076 | 0.074 | 26 |
| 3 | 1x10^6^ | 1x10^7^ | -7786.4 | 17.073 | 0.074 | 27 |
| 3 | 1x10^6^ | 1x10^7^ | -7786.7 | 17.091 | 0.074 | 28 |
| 3 | 1x10^6^ | 1x10^7^ | -7786.6 | 17.085 | 0.075 | 29 |
| 3 | 1x10^6^ | 1x10^7^ | -7786.6 | 17.079 | 0.074 | 30 |
| 4 | 1x10^6^ | 1x10^7^ | -7809.6 | 23.696 | 0.066 | 31 |
| 4 | 1x10^6^ | 1x10^7^ | -7807.6 | 23.618 | 0.066 | 32 |
| 4 | 1x10^6^ | 1x10^7^ | -7801.5 | 23.367 | 0.066 | 33 |
| 4 | 1x10^6^ | 1x10^7^ | -7819.3 | 24.083 | 0.066 | 34 |
| 4 | 1x10^6^ | 1x10^7^ | -7813.5 | 23.847 | 0.066 | 35 |
| 4 | 1x10^6^ | 1x10^7^ | -7802.9 | 23.431 | 0.067 | 36 |
| 4 | 1x10^6^ | 1x10^7^ | -7805.7 | 23.533 | 0.066 | 37 |
| 4 | 1x10^6^ | 1x10^7^ | -7832.1 | 24.593 | 0.066 | 38 |
| 4 | 1x10^6^ | 1x10^7^ | -7803.3 | 23.431 | 0.066 | 39 |
| 4 | 1x10^6^ | 1x10^7^ | -7804 | 23.473 | 0.066 | 40 |
| 5 | 1x10^6^ | 1x10^7^ | -7749.7 | 25.330 | 0.060 | 41 |
| 5 | 1x10^6^ | 1x10^7^ | -7760.8 | 25.747 | 0.060 | 42 |
| 5 | 1x10^6^ | 1x10^7^ | -7752 | 25.418 | 0.060 | 43 |
| 5 | 1x10^6^ | 1x10^7^ | -7750.3 | 25.361 | 0.060 | 44 |
| 5 | 1x10^6^ | 1x10^7^ | -7752.3 | 25.432 | 0.060 | 45 |
| 5 | 1x10^6^ | 1x10^7^ | -7753.9 | 25.485 | 0.060 | 46 |
| 5 | 1x10^6^ | 1x10^7^ | -7754 | 25.493 | 0.060 | 47 |
| 5 | 1x10^6^ | 1x10^7^ | -7751.9 | 25.413 | 0.060 | 48 |
| 5 | 1x10^6^ | 1x10^7^ | -7750 | 25.340 | 0.060 | 49 |
| 5 | 1x10^6^ | 1x10^7^ | -7751.2 | 25.389 | 0.060 | 50 |
| 6 | 1x10^6^ | 1x10^7^ | -7759 | 28.590 | 0.057 | 51 |
| 6 | 1x10^6^ | 1x10^7^ | -7776.4 | 29.174 | 0.057 | 52 |
| 6 | 1x10^6^ | 1x10^7^ | -7768.4 | 28.896 | 0.057 | 53 |
| 6 | 1x10^6^ | 1x10^7^ | -7773.8 | 29.081 | 0.058 | 54 |
| 6 | 1x10^6^ | 1x10^7^ | -7763.7 | 28.747 | 0.057 | 55 |
| 6 | 1x10^6^ | 1x10^7^ | -7749.5 | 28.277 | 0.058 | 56 |
| 6 | 1x10^6^ | 1x10^7^ | -7770.6 | 28.978 | 0.057 | 57 |
| 6 | 1x10^6^ | 1x10^7^ | -7751.9 | 28.350 | 0.057 | 58 |
| 6 | 1x10^6^ | 1x10^7^ | -7767.9 | 28.893 | 0.058 | 59 |
| 6 | 1x10^6^ | 1x10^7^ | -7767.1 | 28.862 | 0.057 | 60 |

Table S2
