# Supplementary material for: Genetic Evidence Highlights Potential Impacts of By-Catch to Cetaceans
Source: PLoS One. 2010 Dec 15;5(12):e15550. doi: 10.1371/journal.pone.0015550 (PMC3002289; doi:10.1371/journal.pone.0015550)
Supplement: Table S3 — Runs for the Bayesian analysis of population structure. Burnin steps maximize the chances of reaching a high probability region in the probability space before the actual estimation. (DOC) [file pone.0015550.s003.doc]

| **k** | **# runs** | **L(K) Mean** | **SD** | **Δ(K) Mean** |
| --- | --- | --- | --- | --- |
| 1 | 10 | -8233.06 | 0.16 | - |
| 2 | 10 | -7943.8 | 2.20 | 59.959 |
| 3 | 10 | **-7786.51** | 0.13 | **1404.618** |
| 4 | 10 | -7809.95 | 9.52 | 8.488 |
| 5 | 10 | -7752.61 | 3.24 | 21.495 |
| 6 | 10 | -7764.83 | 8.90 | - |

Table S3.
