# Supplementary material for: Genetic Evidence Highlights Potential Impacts of By-Catch to Cetaceans
Source: PLoS One. 2010 Dec 15;5(12):e15550. doi: 10.1371/journal.pone.0015550 (PMC3002289; doi:10.1371/journal.pone.0015550)
Supplement: Table S4 — Runs for the Bayesian analysis of population structure. Burnin steps maximize the chances of reaching a high probability region in the probability space before the actual estimation. (DOC) [file pone.0015550.s004.doc]

|  |  | **Allele frequencies** |  |  |  |
| --- | --- | --- | --- | --- | --- |
| **Population** | **Simulation** | **Equidistant** | **Triangular** | **Random** | **Sample** |
| BSS | 1 | 2.7 | 6.8 | 10 | 30 |
|  | 2 | 2.5 | 6.4 | 7.7 | 29 |
|  | 3 | 3.2 | 7.5 | 8.6 | 29 |
|  | 4 | 2.8 | 6.7 | 8.2 | 33 |
|  | 5 | 2.7 | 6.2 | 8.1 | 29 |
|  | 6 | 3 | 5.9 | 8.4 | 29 |
|  | 7 | 2.6 | 6.8 | 10 | 30 |
|  | 8 | 3.6 | 6.5 | 10 | 30 |
|  | 9 | 2.8 | 6.4 | 8.8 | 29 |
|  | 10 | 3 | 6.1 | 8.6 | 30 |
|  | mean | 2.89 | 6.53 | 8.84 | 29.8 |
|  |  |  |  |  |  |
| CSA | 1 | 2.5 | 7.1 | 8.6 | 28 |
|  | 2 | 2.8 | 6.6 | 7.5 | 30 |
|  | 3 | 2.6 | 5.8 | 7.6 | 26 |
|  | 4 | 2.6 | 6.6 | 6.6 | 30 |
|  | 5 | 3.4 | 7.2 | 7.5 | 28 |
|  | 6 | 2.9 | 6.4 | 7.2 | 25 |
|  | 7 | 3.1 | 7.8 | 7.5 | 22 |
|  | 8 | 2.9 | 5.9 | 8.2 | 21 |
|  | 9 | 2.4 | 5.6 | 9 | 20 |
|  | 10 | 2.8 | 6.7 | 7.6 | 20 |
|  | mean | 2.8 | 6.57 | 7.73 | 25 |
|  |  |  |  |  |  |
| BASS | 1 | 3 | 6.1 | 10 | 34 |
|  | 2 | 2.5 | 7.2 | 10 | 32 |
|  | 3 | 2.4 | 7 | 8.1 | 30 |
|  | 4 | 2.8 | 6.6 | 6.8 | 30 |
|  | 5 | 2.8 | 6.6 | 8 | 32 |
|  | 6 | 2.4 | 7 | 7.8 | 30 |
|  | 7 | 2.3 | 7 | 8.1 | 33 |
|  | 8 | 2.8 | 7 | 8.7 | 31 |
|  | 9 | 3 | 6.2 | 8 | 29 |
|  | 10 | 2.3 | 6.8 | 8.5 | 32 |
|  | mean | 2.63 | 6.75 | 8.4 | 31.3 |

Table S4.
