# Supplementary material for: Genetic Evidence Highlights Potential Impacts of By-Catch to Cetaceans
Source: PLoS One. 2010 Dec 15;5(12):e15550. doi: 10.1371/journal.pone.0015550 (PMC3002289; doi:10.1371/journal.pone.0015550)
Supplement: Text S1 — Supporting Text. (DOC) [file pone.0015550.s005.doc]

**Supporting information:** Genetic evidence highlights potential impacts of by-catch to cetaceans

Martin Mendez, Howard C. Rosenbaum, Randall Wells, Andrew Stamper, and Pablo Bordino.

*Sample collection and DNA extraction*

Tissue samples of 245 individuals were obtained from incidentally entangled (by-caught) franciscana dolphins (*Pontoporia blainvillei*) in coastal fishery gillnets in Argentina between 2000 and 2009. These samples were collected as part of a necropsy procedure and were preserved in ethanol (96% v/v). In addition, four pairs and a group of three individuals were captured and released between 2006 and 2008 in locations BSS and BASS [1].

Total genomic DNA was extracted from all tissue samples following the procedures in the QIAamp Tissue Kit (QiaGen, Valencia, CA). For all samples, a fragment of 560 bp of the mtDNA control region was amplified (primers L159256 and H00651) [2], and cycle-sequenced using conditions detailed in Mendez *et al.* (2008) (available from the authors upon request). The samples were then cleaned by filtration in a matrix of Sephadex/water or alternatively by ethanol precipitation, and all were analyzed in a 3730xl DNA Analyzer (Applied Biosystems, Inc. [ABI], Foster City, CA).

Twelve microsatellite loci previously developed for other cetacean species were optimized and amplified for all samples. Each forward primer was modified adding an M13 sequence tail to its 5' end for fluorescent labeling (labeled M13 primer) [3]. PCRs were performed in a 25 µl reaction volume, consisting of 0.25 mM Tris-HCl, 1.25 mM KCl, 0.0375 mM MgCl_2_, 0.03 mM each dNTP, 0.04 µM forward primer, 0.4 µM reverse primer, 0.18 µM labeled M13 primer, 1 U of AmpliTaq DNA Polymerase (ABI), and approximately 5-10 ng of genomic DNA. Thermal profiles for the different loci were adapted from the original amplification conditions [4,5,6,7,8] and are reported in Table S1.

*mtDNA - Haplotyping and diversity estimates*

DNA sequence variation was characterized into mtDNA haplotype definitions following the nomenclature that was developed sequentially in Secchi *et al.* [9], Lazaro *et al.* [10] and Mendez *et al.* (2008). The 560 bp mtDNA fragment was truncated to a 407 bp consensus region containing about 95% of the variation, in order to integrate the shorter sequences obtained from Genbank into our total dataset. Matching of sequences to haplotypes was done using COLLAPSE v1.2 (available from [http://darwin.uvigo.es](http://darwin.uvigo.es/)) and DnaSP v5.0 [11]. We further verified this haplotyping procedure with MacClade v4.01.

*Microsatellite data – Genotyping and diversity estimates*

Microsatellite genotyping was done using the GeneMapper v4.0 software (ABI). Degraded samples, biopsy samples, and those collected from sampling sites with small overall sample size were amplified and typed in duplicates to minimize typing error. Genotype error was evaluated for the remaining samples by re-amplifying and re-typing 10% of the total chosen at random. Overall, 11 cases of allele dropout were detected in our samples, which were solved by triplicate genotyping. GENEPOP v4.0 [12] was used to evaluate linkage disequilibrium (LD) between all pairs of loci for each population (1000 dememorization iterations, 1000 batches, 10000 iterations per batch). Significance levels (*p*=0.05) for departure from Hardy-Weinberg equilibrium (HWE) and for LD were corrected for multiple comparisons with the sequential Bonferroni correction [13].

*Analysis of population structure*

Because allele frequencies are needed for estimations of relatedness, population structure can influence such estimations [14,15]. We previously showed significant population structure for this species both at a regional and local spatial scale. A first evaluation based on the mtDNA control region and encompassing the entire species distribution showed strong genetic structure between areas in Brazil, Uruguay and Argentina, and subpopulation structure within the latter country [16]. Here we use microsatellite and mtDNA sequence data to test for population structure between the two sampling sites (BSS n=89, CSA n=104) where the multiple entanglements took place, prior to the relatedness assessments (multiple entanglements were removed to perform these tests).

Spatial structure of the mitochondrial dataset among the putative populations was evaluated through the Analysis of Molecular Variance procedure [17] as implemented in Arlequin. *F_ST_* and *Φ_ST_* statistics were computed [18]. The significance of the observed *Φ*- or *F*-statistics was tested using the null distribution generated from 10,000 non-parametric random permutations of the data matrix variables.

In order to assess the degree of partitioning in our total sample without *a priori* definition of putative populations, a Bayesian clustering algorithm with Markov Chain Monte Carlo (MCMC) optimization was utilized as implemented in STRUCTURE v2.3.1 [19,20,21]. Given the number of genetic clusters (*K*) as a prior hypothesis, and under the assumption of Hardy-Weinberg and linkage equilibrium within clusters, the algorithm estimates allele frequencies and cluster memberships for all individuals in the total sample, and the log-likelihood of the data for the pre-defined *K* values. We used the admixture model for our inferences, which assumes that individuals have mixed ancestry (i.e. they inherited a fraction of their genome from some ancestors in cluster *K*). Although STRUCTURE allows for the incorporation of sampling location priors, we did not include such information in our models, making them more stringent. The most likely number of clusters for the total sample can then be evaluated by different types of comparisons of the log-likelihood outputs for the different *K* values [22]. We followed a heuristic method to evaluate 1≤*K*≤6 under the admixture model, performing between 10 independent runs (10^6^ burn-in steps, 10^7^ total steps) for *each* value of *K*, for a total of 60 runs (Table S2). The maximum log-likelihood values from all runs corresponding to each given *K* are then averaged, with the corresponding calculation of standard deviation for the averaged group. The most likely number of clusters that better explains our microsatellite dataset then results from the *K* with highest averaged maximum log-likelihood, or the one at which the log-likelihood values rich a plateau. We corroborated this by applying the approach of *Δ(K)* [22] (Table S3).

*Analysis of pedigree relationships*

Hypotheses of pedigree relationships between pairs of individuals were evaluated with the software KINGROUP v.2.0.8. [23]. Relatedness estimations for each pair of simultaneously entangled dolphins were performed within their respective population of origin, identified with the previous analyses of population structure. First, we evaluated the performance of the most commonly used relatedness estimators for our dataset, r_QG_ [24], r_LR_ [25], r_W_ [26], and r_ML_ [23]. We used a Monte Carlo simulation approach to estimate sample mean and variances of relatedness measures for known relationships [27]. We used KINGROUP to generate 100 pairs of individuals for four possible relationships: PO, FS, HS, and U for each of the two populations, using our empirical allele frequencies at these populations. The relatedness of each simulated pair was then calculated in KINGROUP using each of the previously mentioned relatedness estimators. We derived sample variance as the standard deviation of the mean relatedness estimate for each simulated dataset at each population, and assessed estimator bias by comparing the simulated to expected relatedness via two-tailed t-tests.

We followed by calculating the relatedness coefficient for each of our pairs of individuals simultaneously entangled with all four relatedness estimators. This procedure first uses a resampling method to reshuffle alleles at each locus (GuoThompson 1992, Biometrics) and assess the significance of the estimation (i.e. whether individuals are more closely related than expected by chance), and follows by providing the actual value of the relatedness coefficient for each estimator.

As a further approach to evaluate alternative hypotheses of relatedness, we attempted to conduct likelihood ratio tests as implemented in KINGROUP. Given the high statistical power needed for these tests, some samples present a high percentage of type II errors, whereby individuals of a certain relationship would not be resolved as such due an insufficient number of alleles and/or loci in the sample. We conducted a simulation exercise to evaluate the amount of information in our sample and, as a result, the appropriateness of the likelihood ratio tests for our dataset. Replicating the number of alleles per loci in our sample populations, we generated allele frequencies with equifrequent, random (between 0 and 1), and triangular distributions. We then used these generated allele frequencies and the actual allele frequencies in our sample populations to simulate groups of 10 pairs of parent-offspring individuals. Finally, we assessed the type II error of the likelihood ratio tests using PO as the primary hypothesis and U, HS or FS as null hypotheses. We repeated this simulation and assessment procedure ten times for each population (Table S4).

References:

1. Bordino P, Wells RS, Stamper MA (2008) Satellite tracking of Franciscana Dolphins *Pontoporia blainvillei* in Argentina: preliminary information on ranging, diving and social patterns. Paper SC/53/IA32 presented to the IWC Scientific Committee, (unpublished).

2. Kocher TD, Thomas WK, Meyer A, Edwards SV, Paabo S, et al. (1989) Dynamics of mitochondrial DNA evolution in animals: amplification and sequencing with conserved primers. Proceedings of the National Academy of Sciences USA 86: 6196-6200.

3. Schuelke M (2000) An economic method for the fluorescent labeling of PCR fragments. Nature Biotechnology 18: 233-234.

4. Krützen M, Sherwin WB, Berggren P, Gales N (2004) Population structure in an inshore cetacean revealed by microsatellite and mtDNA analysis: Bottlenose dolphins (*Tursiops* sp.) in Shark Bay, Western Australia. Marine Mammal Science 20: 28-47.

5. Valsecchi E, Amos W (1996) Microsatellite markers for the study of cetacean populations. Molecular Ecology 5: 151-156.

6. Buchanan FC, Friesen MK, Littlejohn RP, Clayton JW (1996) Microsatellites from the beluga whale *Delphinapterus leucas*. Molecular Ecology 5: 571-575.

7. Hoelzel AR (1998) Genetic structure of cetacean populations in sympatry, parapatry, and mixed assemblages: Implications for conservation policy. Journal of Heredity 89: 451-458.

8. Shinohara M, Domingo-Roura X, Takenaka O (1997) Microsatellites in the bottlenose dolphin *Tursiops truncatus*. Molecular Ecology 6: 695-696.

9. Secchi ER, Wang JY, Murray BW, Rocha Campos CC, White BN (1998) Population differentiation in the franciscana (*Pontoporia blainvillei*) from two geographic locations in Brazil as determined from mitochondrial DNA control region sequences. Canadian Journal of Zoology 76: 1622-1627.

10. Lazaro M, Lessa EP, Hamilton H (2004) Geographic genetic structure in the franciscana dolphin (*Pontoporia blainvillei*). Marine Mammal Science 20: 201-214.

11. Rozas J, Sánchez-DelBarrio, J. C., Messegyer, X. and Rozas, R. (2003) DnaSP, DNA polymorphism analyses by the coalescent and other methods. Bioinformatics 2496-2497.

12. Rousset F (2008) GENEPOP ' 007: a complete re-implementation of the GENEPOP software for Windows and Linux. Molecular Ecology Resources 8: 103-106.

13. Rice WR (1989) Analyzing tables of statistical tests. Evolution 43: 223-225.

14. Weir BS, Anderson AD, Hepler AB (2006) Genetic relatedness analysis: modern data and new challenges. Nature Reviews Genetics 7: 771-780.

15. Fernandez J, Toro MA (2006) A new method to estimate relatedness from molecular markers. Molecular Ecology 15: 1657-1667.

16. Mendez M, Rosenbaum HC, Bordino P (2008) Conservation genetics of the franciscana dolphin in Northern Argentina: population structure, by-catch impacts, and management implications. Conservation Genetics 9: 419-435.

17. Excoffier L, Smouse PE, Quattro JM (1992) Analysis of molecular variance inferred from metric distances among DNA haplotypes: application to human mitochondrial DNA restriction data. Genetics 131: 479-491.

18. Weir BS, Cockerham CC (1984) Estimating F-statistics for the analysis of population structure. Evolution 38: 1358-1370.

19. Pritchard JK, Stephens M, Donnelly P (2000) Inference of population structure using multilocus genotype data. Genetics 155: 945-959.

20. Hubisz MJ, Falush D, Stephens M, Pritchard JK (2009) Inferring weak population structure with the assistance of sample group information. Molecular Ecology Resources 9: 1322-1332.

21. Falush D, Stephens M, Pritchard JK (2003) Inference of population structure using multilocus genotype data: linked loci and correlated allele frequencies. Genetics 164: 1567-1587.

22. Evanno G, Regnaut S, Goudet J (2005) Detecting the number of clusters of individuals using the software structure: a simulation study. Molecular Ecology 14: 2611-2620.

23. Konovalov DA, Manning C, Henshaw MT (2004) KINGROUP: a program for pedigree relationship reconstruction and kin group assignments using genetic markers. Molecular Ecology Notes 4: 779-782.

24. Queller DC, Goodnight KF (1989) Estimating relatedness using genetic markers. Evolution 43: 258-275.

25. Lynch M, Ritland K (1999) Estimation of Pairwise Relatedness With Molecular Markers. Genetics 152: 1753-1766.

26. Wang J (2002) An estimator for pairwise relatedness using molecular markers. Genetics 160: 1203-1215.

27. Van de Casteele T, Galbusera P, Matthysen E (2001) A comparison of microsatellite-based pairwise relatedness estimators. Molecular Ecology 10: 1539-1549.
